# Branch Retinal Artery Occlusion following Dental Extraction

**DOI:** 10.1155/2014/202834

**Published:** 2014-12-16

**Authors:** Tevfik Oğurel, Zafer Onaran, Reyhan Oğurel, Nurgül Örnek, Nesrin Büyüktortop Gökçınar, Kemal Örnek

**Affiliations:** Department of Ophthalmology, Kırıkkale University Medical Faculty, Kırıkkale University Campus, Ankara Road 6 Km, Yahsihan, 71100 Kırıkkale, Turkey

## Abstract

*Aim*. To describe a case of branch retinal artery occlusion following dental extraction and to point out the ophthalmic complications of dental procedures to ophthalmologists and dentists. *Case*. A 51-year-old woman was referred to our clinic with painless sudden visual loss in her left eye after tooth extraction two days ago. In her left eye the best corrected visual acuity was 6/30 and fundus examination revealed peripapillary flame-shaped hemorrhages and pale retina in the upper temporal arcuate. The right eye examination was unremarkable. *Conclusion*. Dental procedures can lead to miscellaneous ophthalmic complications possibly due to the close proximity of the anatomic structures. Retinal arterial occlusion is a rare but serious cause of permanent visual loss among these dental procedures where the exact pathologic mechanism is still obscure.

## 1. Introduction

Dental procedures rarely induce ophthalmic complications including diplopia, strabismus, ptosis, and amaurosis fugax [1]. Injection of anesthetic solution into the oral cavity is the leading factor in the development of these complications. Besides this, tooth extraction is also charged for ocular complications. Since mentioned ones are often transient, more catastrophic complications like permanent amaurosis, orbital cellulitis, orbital abscess, and endophthalmitis were reported in the literature [2]. Dental procedures under not only local anesthesia but also general anesthesia were shown to cause visual disturbances [3].

Here we report a case of visual loss due to branch retinal artery occlusion after dental extraction in a middle-aged patient.

## 2. Case Report

**Figure 1:**
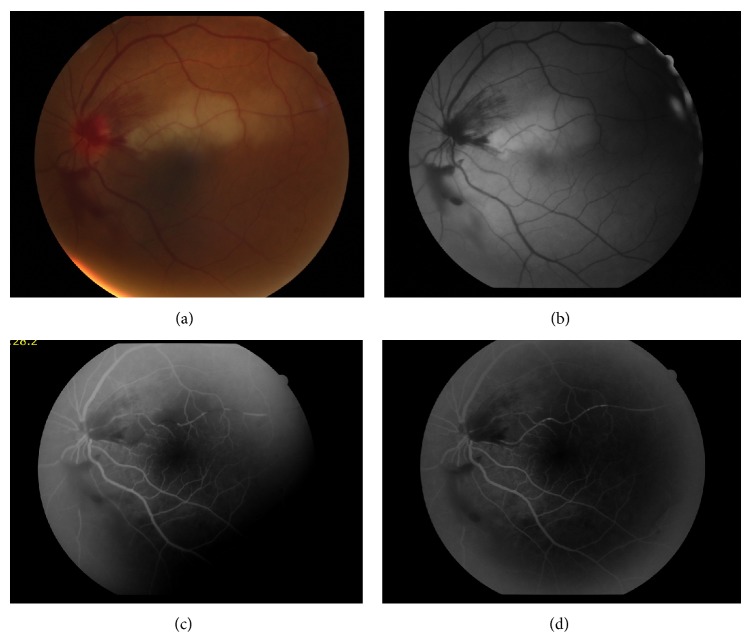
(a and b) Color and red free fundus photograph of the left eye representing peripapillary flame-shaped hemorrhages and pale retina in the upper temporal arcuate. (c and d) Fundus fluorescein angiography showed filling defects in the upper temporal branch retinal artery in the early phase. There were also multiple embolic opacities along the artery.

A 51-year-old woman was referred to our clinic with painless sudden visual loss in her left eye who was otherwise healthy except for arterial hypertension. Two days ago she was evaluated for extraction of third molar teeth. Four-five hours after tooth extraction she realized blurred vision in her left eye. On initial ophthalmic examination, the best corrected visual acuity was 6/6 in the right eye and 6/30 in the left eye. Slit-lamp examination was unremarkable except for a relative afferent pupillary defect (RAPD) in the left eye. Fundus examination revealed peripapillary flame-shaped hemorrhages and pale retina in the upper temporal arcuate in the left eye (Figures 1(a)-1(b)). Visual fields revealed an inferior altitudinal defect OS. Fundus fluorescein angiography showed filling defects in the upper temporal branch retinal artery in the early phase (Figure 1(c)). There were also multiple embolic opacities along the artery (Figure 1(d)). Optic coherence tomography imaging showed increased retinal thickness due to edema caused by ischemia. Laboratory investigations including C-reactive protein (CRP) and sedimentation were found to be normal and it was learned that lidocaine hydrochloride with epinephrine was used for dental anesthesia from her dentist.

Visual acuity of the left eye was found to be unchanged at the first month follow-up.

## 3. Discussion

The central retinal artery, a branch of the ophthalmic artery, divides into multiple branches at the level of the optic nerve head. Perfusion of inner retinal layers corrupts when these arterial branches occluded commonly by an embolus. The most common reasons include cholesterol emboli from aortocarotid atheromatous plaques, platelet-fibrin emboli from thrombotic disease, and calcific emboli from cardiac valvular disease.

There is no consensus on the exact etiology of vascular ocular complications after dental procedure. Reported cases in the literature frequently developed after intraoral dental anesthesia but the mechanism is still not fully understood [4]. However, it is generally agreed that the local anesthetic solution reaches the orbit through vascular, neurological, or lymphatic network [5].

According to a hypothesis the proposed mechanism of occlusion of the artery begins with intra-arterial administration of the anesthetic solution and reaches the eye following the maxillary artery/inferior alveolar artery, middle meningeal artery, and ophthalmic artery route. Amaurosis, impaired vision, and loss of the pupillary light reflex are the clinical features of this situation. Once the anesthetic reaches the retinal artery, reflective vasospasm of the central retinal artery induced by either the active ingredient or the epinephrine could result in ischemia and necrosis of the retinal tissue [6]. However, this mechanism can be applied on central retinal artery and is not suitable for explanation of the branch retinal artery occlusion presented in our case.

Another hypothesis is the embolic occlusion of the retinal artery. The nature of the emboli could be fat, air, fluid, or calcific. Rishiraj et al. reported a case of central retinal artery occlusion in a 74-year-old man that resulted in loss of vision in one eye [2]. They have shown fluidic embolization of the entire retinal artery tree where local anesthesia was administered containing prilocaine. Another permanent blindness caused by retinal artery occlusion occurred after dental anesthesia of upper teeth that associated with injection of local anesthetic solution containing procaine hydrochloride was described by Walsh [5].

The accompanying retinal hemorrhages around the optic nerve head are also complicating our case which is also hard to explain. Incipient occlusion of the central retinal vein or a Valsalva maneuver during dental procedure might lead to these hemorrhages. Differential diagnosis includes hypertensive retinopathy, nonarteritic anterior ischemic optic neuropathy (NAION), and vasculitic occlusion including giant cell arteritis. Unilateral involvement and controlled hypertension in our case are not compatible with hypertensive retinopathy. Also normal levels of sedimentation rates and CRP made the vasculitis diagnosis less possible. Presentation of NAION commonly in the 6th or 7th decade, accompanying systemic diseases including diabetes mellitus and hyperlipidaemia, and presence of hyperemic disc swelling were the factors that are not compatible with our case.

Apart from the aforementioned hypotheses about the source of the emboli causing arterial occlusion, emboli could be originated from the carotid artery system induced by Valsalva and cervical hyperextension during dental procedure so it may not be directly associated with local anesthetic injection or dental extraction.

Despite the obscurity of the pathologic mechanism causing arterial occlusion, avoiding intravascular injection seems to be the only preventive effort. Anesthetic agents including epinephrine should also be used carefully in patients with cardiovascular disease and hypertension. Awareness of this complication and urgent referral to an ophthalmologist may give a chance in the mean of reversal of the ischemic retinal damage where the minutes count.
